# Visual perspective taking neural processing in forensic cases with high density EEG

**DOI:** 10.1038/s41598-024-66522-y

**Published:** 2024-07-10

**Authors:** Vincent Rochas, Marie-Louise Montandon, Cristelle Rodriguez, François R. Herrmann, Ariel Eytan, Alan J. Pegna, Christoph M. Michel, Panteleimon Giannakopoulos

**Affiliations:** 1 Functional Brain Mapping Laboratory, Department of Basic Neurosciences, University of Geneva, Geneva, Switzerland; 2 Human Neuroscience Platform, Fondation Campus Biotech Geneva, Geneva, Switzerland; 3 Department of Rehabilitation and Geriatrics, Geneva University Hospitals and University of Geneva, Geneva, Switzerland; 4 Division of Institutional Measures, Medical Direction, Geneva University Hospitals, Geneva, Switzerland; 5 Faculty of Medicine of the University of Geneva, Geneva, Switzerland; 6 School of Psychology, University of Queensland, Brisbane, Australia

**Keywords:** Empathy, Perception

## Abstract

This EEG study aims at dissecting the differences in the activation of neural generators between borderline personality disorder patients with court-ordered measures (BDL-COM) and healthy controls in visual perspective taking. We focused on the distinction between mentalizing (Avatar) and non-mentalizing (Arrow) stimuli as well as self versus other-perspective in the dot perspective task (dPT) in a sample of 15 BDL-COM cases and 54 controls, all of male gender. BDL-COM patients showed a late and diffuse right hemisphere involvement of neural generators contrasting with the occipitofrontal topography observed in controls. For Avatars only and compared to controls, the adoption of Self perspective involved a lower EEG activity in the left inferior frontal, right middle temporal cortex and insula in BDL-COM patients prior to 80 ms post-stimulus. When taking the Other-perspective, BDL-COM patients also showed a lower activation of superior frontal, right inferior temporal and fusiform cortex within the same time frame. The beta oscillation power was significantly lower in BDL-COM patients than controls between 400 and 1300 ms post stimulus in the Avatar-Other condition. These results indicate that BDL-COM patients display both altered topography of EEG activation patterns and reduced abilities to mobilize beta oscillations during the treatment of mentalistic stimuli in dPT.

## Introduction

Empathy is a key notion of social psychology that determines our ability to promote human interactions, professional and intimate relationships. It includes both an affective dimension referring to the automatic sharing of emotions and a cognitive component close to the notion of theory of mind (ToM) that encompasses the ability to judge the other’s viewpoints, wishes and hidden or explicit intentions with subsequent adaption of the behavior^1–5^. Previous studies postulated the existence of an explicit part of the ToM based on the logical analysis of other’s emotions and thoughts and an implicit part that corresponds to a rapid and mostly unconscious taking into account of other’s viewpoints even when not needed for task processing^6–13^. This latter may be critical for mindreading—the ability to predict and interpret the behavior of others in terms of their underlying mental states in order to inhibit automatically socially inappropriate reactions and protect close relationships^14^.

The neural substrates of automatic perspective taking have been often analyzed using the dot perspective-taking task (dPT), originally developed by Samson and colleagues^10^. In this task, participants are asked to count the number of dots on a screen. An Avatar is also present on the screen and sees a number of dots that is either the same or less than the number of dots that the participant sees. The left temporoparietal junction (TPJ) /inferior parietal cortex as well as the bilateral inferior frontal gyrus (for inhibiting one’s own perspective) are usually activated in visual perspective-taking paradigms. A recent review by Bukowski^15^ further confirmed the involvement of frontal lobe subdivisions (dorsolateral prefrontal cortex, posterior middle, and inferior frontal gyrus), dorsal precuneus, and temporoparietal junction, as well as additional activation of the inferior parietal sulcus, inferior posterior temporal cortex, and superior cerebellum in the dPT. However, it is still debatable whether these brain activation patterns correspond to implicit mentalizing that depends on with the human nature of the Avatar or attention-orienting processes that could be present as a response to the stimulus not involving mentalization processes (for review^14,16–19^). In a first fMRI study, Schurz et colleagues^20^ reported a spontaneous activation in right temporo-parietal junction, ventral prefrontal cortex and ventral precuneus during self-perspective judgments when using an Avatar (mentalistic) but not an Arrow (non-mentalistic control) that was taken to reflect implicit processing of information linked to the other’s perspective during Self condition. This viewpoint has been challenged by neurostimulation reports, which show that transcranial magnetic stimulation of right TPJ impairs performance on all self-perspective trials (Arrow and Avatar), indicating the predominance of attentional processes rather than implicit mentalization^17,21^. More recently, our fMRI data supported the idea that mentalistic stimuli (Avatars) induce a distinct pattern of brain activation compared to non-mentalistic ones (Arrows), including parts of the mentalizing and salience networks and, to a lesser extent, attentional and cognitive control-related areas^22^.

Despite the fact that classical EEG recordings have the advantage to monitor the neural responses with high temporal precision, such contributions in dPT remain scarce. McCleery and colleagues^23^ reported that the temporoparietal cortex is involved in the distinction between Self and Other-perspectives, whereas the right frontal cortex resolves the conflict between Self and Other-perspectives. Ferguson and colleagues^24^ reported that the amplitudes of P100, P200, P300, and late frontal slow wave (LFSW) ERP components were reduced when a child Avatar was used pointing to the presence of mentalizing and non-mentalizing attentional processes in visual perspective-taking. In a recent study of 39 healthy controls, we examined in detail the EEG activation patterns in dPT task. Besides the increased activation of neural sources when treating mentalistic (Avatar) versus non-mentalistic (Arrow) stimuli, we demonstrated a mentalistic-related temporal shifting of brain generators from posterior visual areas to anterior frontal areas. When taking the Other’s perspective, there was an increased recruitment of generators in the occipital and temporal lobes and later on in mentalizing and salience networks bilaterally before spreading to right frontal lobe subdivisions. Moreover, increased beta oscillations in the Other-perspective occurred for the Avatar condition only over occipito-parietal, then frontal areas^25^.

ToM and in particular perspective taking is severely affected in a variety of psychiatric disorders including autism, schizophrenia, bipolar disorder and attention-deficit/hyperactivity disorder^26–32^. Among these conditions, borderline (BDL) and antisocial personality disorders are known to present early and severe deficits in perspective taking that may determine impaired self-referential processing, moral cognition and law violations^33,34^. BDL patients suffer from severe disturbances in perspective taking and do not often integrate what they believe others think about them into their self-concept^32,35^. In the same line, recent contributions revealed that antisocial patients display decreased mentalization abilities as well as impaired functional connectivity in social cognition network in perspective taking^36–38^. Using the dPT task, Drayton and colleagues^39^ reported that psychopaths are able to represent others’ perspective in goal-conducive tasks but show a striking ability of ignoring it in nongoal-relevant situations. They postulated that their lack of ability to automatically represent the belief states of others when it does not serve their own ends may be at the origin of their maladaptive social behavior.

The present report explores the differences in the EEG activation of neural generators between forensic cases with BDL diagnosis and court-ordered measures for criminal offenses (BDL-COM) and age matched healthy controls without history of previous convictions. As in our previous study^25^, we used different steps of neural processing focusing on the distinction between mentalizing and non-mentalizing stimuli with high-density EEG recordings at the surface and in the inverse space to define the brain sources of electrical activity, and time–frequency decomposition to explore the dynamic changes in amplitude and phase of neural oscillations. Our main hypothesis is that BDL-COM patients display EEG deficits in the activation of neural generators involved in perspective taking. In particular, we expected significant decreases in mentalistic-related activation of anterior cortical areas as well as beta oscillation activity, observed in occipito-temporal and frontal areas when taking the other position, in these cases compared to healthy controls. In order to test our hypotheses, we included the comparison of EEG activation patterns using Arrow versus Avatar according to the perspective taken (Self vs. Other-perspective). To simplify the experimental design and according to Saether and colleagues^40^, we limited the present analysis to inconsistent trials (conflict between Self- and Other-perspective).

## Materials and methods

### Ethical statements

The study was reviewed and approved by the local Ethics Committee [Commission cantonale d’éthique de la recherche (CCER)]. The participants provided their written informed consent prior to inclusion. All control cases were recruited via advertisements in local newspapers and media. The 15 patients were recruited among those regularly followed-up for COM in the Service of Institutional Measures, a specialized division in charge of COM in University Hospitals of Geneva. All methods were performed in accordance with the World Medical Association Declaration of Helsinki and the principles of Good Clinical Practice.

### Participants

All of the participants performed a detailed psychiatric assessment made by a board-certified fully trained psychiatrist (AE). The exclusion of acute psychiatric disorders was confirmed by the Mini Neuropsychiatric Interview^41^. Cases were also excluded if they had an history of loss of consciousness lasting longer than 30 min, head injury or post-concussion symptoms, auditory or visual deficits, seizure and neurological disorders, as well as regular use of psychotropic medication.

As part of their initial evaluation, both controls and BDL-COM patients were assessed using the following tools:

HCR-20 (Historical Clinical Risk 20) version 2 is composed of one static and two dynamic clinician reported scales. This scale is a wide range violence assessment tool developed by Webster, Evan, Douglas, and Witrup in 1995 using a sample of institutionalized people who were followed for approximately 2 years after their discharge into the community^42^. HCR-20 consists of 20 items and assesses past, present and future indicators of violence. It contains 20 items: 10 related to historic items (previous violence, age at first violent incident, relationship instability, employment problems, substance use problems, major mental illness, psychopathology, early maladjustment, personality disorder and prior supervision failure; five clinical items (lack of insight, negative attitudes, active symptoms of major mental illness, impulsivity and unresponsiveness to treatment); and five ‘risk management’ items (plans lack feasibility, exposure to destabilizes, lack of personal support, non-compliance with remediation attempts and stress)^43,44^.

PCL-R (Psychopathy Checklist Revised) is a pivotal tool to identify psychopathic individuals in correctional settings. In 1991 Hare designed this scale to measure the clinical construct of psychopathy, and since it has become the leading instrument to predict recidivism, violence and treatment outcome^45,46^.

The Wechsler Adult Intelligence Scale (WAIS)^47^ is a general intelligence battery used to evaluate patient’s intelligence quotient (IQ). The ten core subtests of the battery give rise to four index scores including the Verbal Comprehension Index, the Perceptual Reasoning Index, the Working Memory Index, and the Processing Speed Index.

The mini-Social cognition and Emotional Assessment (SEA) is a quick clinical tool that assesses ToM and emotion recognition deficits. One part is a reduced and modified version of the faux-pas test, and the second part is a reduced version of Paul Ekman emotion recognition test^48^, resulting in two computational scores (ToM and emotion recognition) and a general composite score. In the shortened faux-pas test, ten stories are presented, including five social faux-pas stories and 5 control stories without any faux-pas. Participants are asked to detect and explain faux-pas as well as to make interferences about intentions, beliefs and feelings of other’s (Was a faux-pas present? What was the faux-pas? Who made it? Why? Was it intentional? How did the victim feel?). In the reduced (35 faces) emotion recognition test, participants identify which emotion was expressed among a list of seven different emotions (fear, sadness, disgust, surprise, anger, happiness, and neutral) depicted in a series of photographs. The two subtests of the mini-SEA result in two computational scores (ToM and emotion recognition) converted to the composite subscores (from 0 to 15, respectively), and in an overall mini-SEA composite score (maximum score of 30) obtained by adding the two composite subscores.

The Geneva Social Cognition Scale (GeSoCS) Is a medium duration assessment tool that detects and characterizes significant changes in social cognition and ToM. It is a 100-point scale composed of 6 subtests: ToM stories, recognition of social emotions, false beliefs, inferences, absurdity judgment, and planning abilities^49^.

The French version of the Toronto Alexithymia Scale (TAS)^50^ is a 20-item instrument that is one of the most commonly used measures of alexithymia. Alexithymia refers to people who have trouble identifying and describing emotions and who tend to minimize emotional experience and focus attention externally. Items are rated using a five-point Likert scale whereby 1 = strongly disagree and 5 = strongly agree. There are 5 items that are negatively keyed (items 4, 5, 10, 18, and 19). The total alexithymia score is the sum of responses to all 20 items. The TAS-20 uses cutoff scoring: equal to or less than 51 = non-alexithymia, equal to or greater than 61 = alexithymia. Scores of 52 − 60 = possible alexithymia.

BDL diagnosis was made according to ICD-10 criteria (World Health Organization. ICD-10: International statistical classification of diseases and related health problems: Tenth revision. 2nd ed. Geneva: World Health Organization), was extracted from the psychiatric expert assessments. Subsequently, it was confirmed by the assessment made by the board-certified psychiatrist. In case of disagreement, the candidate cases were excluded from further investigations.

### Dot perspective taking task

**Figure 1:**
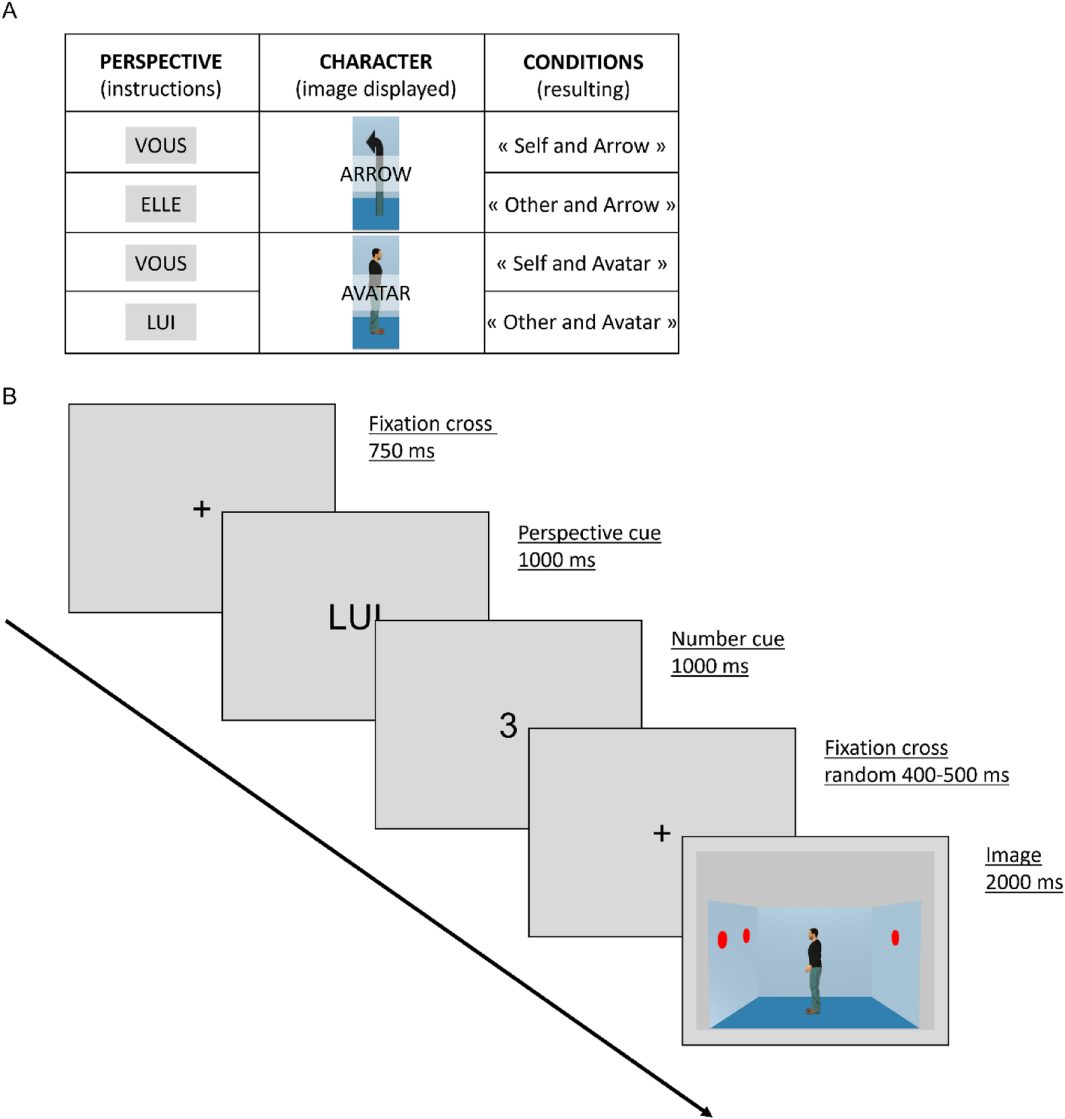
Schematic representation of the task. (**A**) The table summarizes the different conditions resulting from the combinations of instructed perspectives and character displayed on the images. (**B**) The lower diagram represents the time course of one trial as displayed on the screen for the participants.

The dPT task used in this study is derived from Samson and colleagues^10^, and was already used in a previous EEG study^25^. The task consists in the presentation of a picture of a scene of an Avatar in the middle of a square room and looking in one direction either left or rightward. There are zero to three red dots distributed on the two side walls of the represented room. Except for a few trials using a no-dot picture, all the trials are inconsistent in terms of the number of dots seen by the participant and by the Avatar in the picture. An equivalent set pictures is displaying an Arrow instead of the Avatar in order to investigate the Character-related process. In total, 96 trials of each Arrow with Self perspective, Arrow with the Other perspective, Avatar with Self perspective and Avatar with Other perspective are displayed (Fig. 1A). In addition, 48 filler trials displaying zero dots and demanding less effort are also used to check for attention of the participants. For each condition there is an equal proportion of correct and incorrect trials. Trials are displayed randomly in 3 distinct blocks of 144 trials in order to allow for little breaks during the task and preserve the participants’ attention.

The task presentation was controlled by E-Prime 3.0 software (Psychology Software Tools, Pittsburgh, PA) and displayed on a LED screen. One trial (see Fig. 1B) starts with a fixation cross for 750 ms, followed by a perspective cue for 1000 ms instructing the perspective that has to be taken by the participant, i.e. himself or the Avatar-Arrow, followed by a number cue for 1000 ms indicating the number of dots to be seen from 0 to 3. After another fixation cross, displayed for a random duration between 400 and 500 ms, the picture of the scene appears for 2000 ms. After the scene display, the participant had to respond if the cued number corresponds to the number of dots actually seen from the cued perspective. The choice between correct or incorrect was delivered through a two button press on a response box with the dominant hand.

### EEG acquisition

High density EEG was recorded in a dark and soundproofed Faraday cage using an EGI 256 electrode Hydrocel Geodesic Sensor Net connected to an EGI GES 400 amplifier with a sampling frequency set to 1000 Hz and referenced to the Cz electrode. The impedance of the electrodes was checked and kept under 30 kOhms during the sessions. Participants placed their chins on a chinrest situated at 80 cm from the screen and were instructed to keep still during the task performance and recording.

### EEG preprocessing

Using freely available software Cartool 3.91 (https://sites.google.com/site/cartoolcommunity/)^51,52^, the channels corresponding to the cheeks and neck electrodes were removed resulting in 204 channels in total. The EEG were filtered with a DC removal (or 0 Hz high pass), a bandpass Butterworth filter from 1 to 80 Hz and a Butterworth notch filter at 50 Hz and all possible harmonics. The recordings were reviewed by an experienced EEG analyst (VR), and the periods containing large movement artefacts and the bad channels were discarded from further analyses. In average 197 channels were used (mean channel number = 197.2; SD = 3.8). The data were then decomposed following an independent component analysis with Matlab using runICA from EEGlab. Based on their activation time courses and topographies, the resulting components identified as non-EEG (eye saccades and blinks, cardiac interference and more rarely from neck or jaw muscle tension) were subtracted from the data for further analyses. Using software Cartool, the initially identified bad channels were reconstructed using 3D spline interpolation.

### Event-related analyses

First, epochs were selected from − 500 to + 1500 ms time locked on the scene picture onset and separated for the four different conditions namely Arrow with Self perspective, Arrow with Other perspective, Avatar with Self perspective and Avatar with Other perspective. For each participant, the number of epochs were equally adjusted between conditions (random picking of the number for the lowest condition). The clean EEG epochs were first averaged per participant per condition in order to compute surface ERPs (Event Related Potentials). The epochs of each participant for all four conditions were also used to compute event related source reconstruction in Cartool^52^ in order to characterize at the source level the differences observed on the surface. The epoch data went through a spatial filtering of the surface signal considering the position of the electrodes on the scalp. The employed inverse model was based on an MNI template head segmented into 4 shells (scalp, skull, CSF, brain), 6008 solution points symmetrically distributed in the grey matter and an EGI net model corresponding to the 204 remaining electrodes co-registered on the template scalp. A lead field was calculated for the segmented template head using Locally Spherical Model with Anatomical Constraints (LSMAC) an exact spherical equation in order to calculate a distributed linear inverse solution LORETA between the 204 electrodes and the 6008 solution points. An individual normalization using the background activity from the results of inverse solution of the whole epoch data was used to estimate a baseline and a scaling factor for each solution point. We obtained individual event related source reconstructions in scalar values for the four different conditions.

### Time frequency decomposition

A time–frequency decomposition of the signal was computed with a fast Morlet transform in MATLAB 2018b on the selected clean epochs from − 500 to + 1500 ms relative to the stimulus onset, reduced to absolute values and averaged across epochs. Event-related spectral perturbation (ERSP) was computed (average of the absolute value of the time frequency decomposition) and the obtained time–frequency series was corrected by subtraction and division by the averaged baseline period from − 400 to − 100 ms (Neuper and Pfurtscheller 2001). Inter-trial phase coherence (ITC) amplitudes—i.e. phase-locked part of the ERSP signal—were also calculated from time–frequency decomposition (average of the complex numbers characteristics by the absolute value), reflecting the phase consistency across trials^53^.

### Statistics

Fisher exact tests were used to compare sociodemographic (age, education) and clinical variables (TAS, PCL-R, HCR-20, WAIS-IV, GeSoCS, and mini-SEA scores) between controls and BDL-COM patients. Correction for multiple comparison in Table 1 was performed using the Benjamini–Hochberg method. Mixed-design ANCOVAs were computed on the behavioral data (percentage of correct responses and reaction times) with the within subject factors identity (Arrow vs. Avatar) and perspective (Self vs. Other), the between subject factor group (patients vs. control participants) and age as confounding explanatory variable. The surface ERPs were loaded in the all-channel randomization statistic toolbox RAGU^54^ (for details on statistical principles^55^). Based on all channels and time points of all conditions and groups, a multidimensional scale determined disparity between participants and was used to define possible outliers, subsequently excluded from further analysis. A topographic consistency test, based on comparison of the grand-mean global field power (GFP) of original data against grand-mean GFP of shuffled maps, was conducted for each condition in order to define a period for which the neural activation across subjects remained consistent. Further event-related analyses were conducted on the period of time showing topographic consistency across all conditions. A three by two ANOVA, comparing differences among factors for original data against condition randomized data, with Arrow-Avatar and Self-Other-perspective as within subject factors and the patients-controls as between subject factor was conducted on the global field power (GFP). A topographic three by two ANOVA with the same factor design was similarly conducted on topographies in order to reveal qualitative differences of neural processing distribution. In order to address the issue of false positives across time, the count of significant time points obtained in the original data was tested against the distribution of significant *p*-values for all randomization runs. In order to characterize the differences obtained at the surface, the event related source reconstructions for the four different conditions were tested between groups using unpaired t-test statistics with a time constraint for at least 3 consecutive significant points and false discovery rate (FDR) correction. For the ERSP and ITC, the analyses of differences in brain oscillations were performed with cluster-based permutation tests between groups for each of the four conditions using a Matlab based Fieldtrip function^56^. The considered period for this analysis was from 0 (excluding the baseline) to 1300 ms (avoiding edge artifact).

**Table 1:** Clinical description of the sample.

	CTL	BDL-COM	Total	P		BH
N	54 (78.3%)	15 (21.7%)	69 (100.0%)			
Education/number of years	14.53 (2.56)	13.27 (4.06)	14.25 (2.96)	0.146		
Age	31.26 (10.18)	45.07 (17.44)	34.26 (13.28)	< 0.001	*	*
Toronto alexithymia scale (TAS)						
Description of feelings (max = 25)	12.04 (3.82)	13.93 (4.51)	12.46 (4.02)	0.108		
Identification of feelings (max = 35)	12.91 (4.44)	17.33 (7.73)	13.88 (5.59)	0.006	*	*
External oriented thought (max = 40)	16.83 (4.47)	16.20 (3.88)	16.69 (4.32)	0.622		
Total score (max = 100)	41.00 (10.49)	47.47 (13.65)	42.41 (11.46)	0.053		
PCL-R						
F1 (max = 16)	0.37 (1.10)	2.67 (3.39)	0.87 (2.06)	< 0.001	*	*
F2 (max = 20)	1.67 (2.66)	4.73 (2.81)	2.33 (2.96)	< 0.001	*	*
Total score (max = 40)	2.39 (3.65)	7.87 (5.26)	3.58 (4.61)	< 0.001	*	*
HCR-20						
Chronological factors (max = 20)	0.70 (1.56)	2.67 (1.76)	1.13 (1.79)	< 0.001	*	*
Clinical factors (max = 10)	0.26 (0.62)	1.33 (0.82)	0.49 (0.80)	< 0.001	*	*
Risk factors (max = 10)	0.11 (0.46)	0.87 (1.06)	0.28 (0.70)	< 0.001	*	*
Total score (max = 40)	1.81 (5.79)	4.87 (2.70)	2.48 (5.41)	0.053		
WAIS-IV						
Verbal comprehension	38.00 (8.29)	35.93 (5.32)	37.55 (7.76)	0.365		
Perceptual reasoning	31.69 (7.56)	32.53 (4.69)	31.87 (7.02)	0.682		
Working memory	22.69 (5.20)	21.73 (4.73)	22.48 (5.08)	0.525		
Processing speed	20.35 (4.67)	20.53 (5.29)	20.39 (4.77)	0.897		
Total score	112.72 (20.43)	110.73 (12.13)	112.29 (18.88)	0.721		
GeSoCS (max = 100)	91.23 (5.21)	90.47 (5.02)	91.07 (5.14)	0.614		
Mini-SEA (max = 30)	27.14 (2.00)	26.38 (1.75)	26.97 (1.96)	0.186		

CTL: controls; BDL-COM: borderline patients with court-ordered measures; PCL-R: psychopathy checklist revised; HCR-20: historical clinical risk 20; WAIS-IV: Wechsler adult intelligence scale-IV; GeSoCS: Geneva social cognition scale; SEA: mini social cognition and emotional assessment.

The adapted Benjamini–Hochberg p threshold was fixed at 0.020455.

## Results

### Descriptive data

BDL-COM patients were significantly older and presented with significantly higher PCL-R and HCR-20 scores compared to controls. Although this is partly expected given the forensic characteristics of our patients, one should note that the levels of psychopathy remained quite modest with PCL-R (Psychopathy Checklist Revised) total scores that were lower than 15 in all of the cases. According to the Toronto Alexithymia Scale (TAS) assessment, the identification of feelings was significantly more difficult in BDL-COM patients compared to controls. In contrast, the two groups were comparable in respect to Wechsler Adult Intelligence Scale IV (WAIS-IV), Geneva Social Cognition Scale (GeSoCS) and mini-Social cognition and Emotional Assessment (SEA) scores with good levels of global intelligence and preserved empathy abilities.

### Behavioral performances

**Figure 2:**
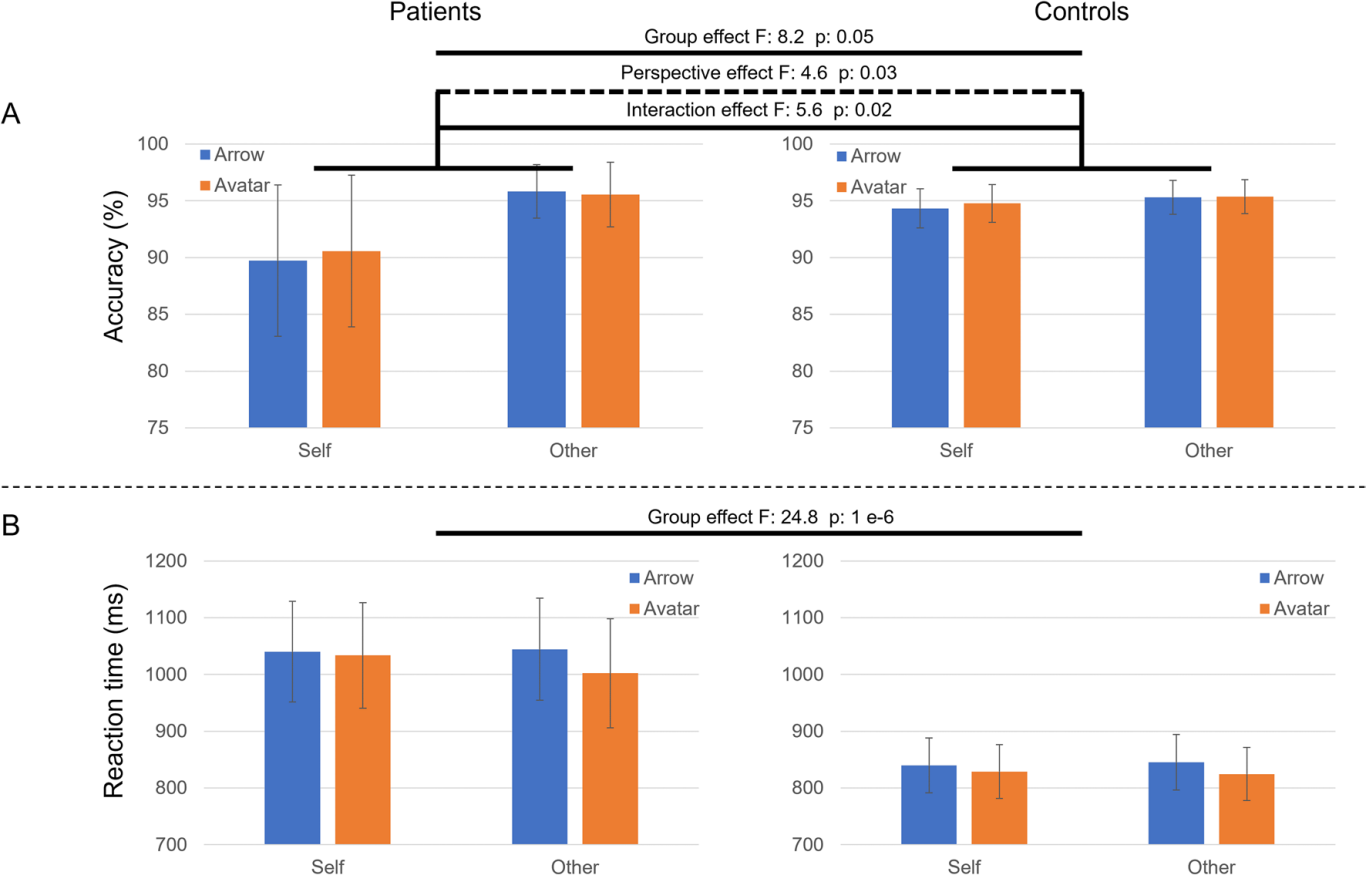
Behavioural metrics. (**A**) The two upper bar plots represent the accuracy in % and their 95% confidence intervals for the patients (left) and controls (right) for the four different conditions of the task (arrow-avatar × self-other-perspective). Similarly, (**B**) the lower bar plots represent the reaction time in ms for the patients (left) and controls (right) for the four different conditions of the task (arrow-avatar × self-other-perspective). Statistically significant results from the mixed-design ANCOVA (character × perspective × group) are also displayed on top of the plots, i.e. main effect of Group and Perspective and their interaction for the accuracy; and main effect of Group for the reaction time.

The demographic and clinical characteristics in the present series are summarized in Table 1. The accuracy and reaction time values are summarized in Table 2. The overall performance was excellent for both BDL-COM patients (92.9%, SD: 10.1%) and controls (95%, SD: 9%). The average reaction time was 1030 ms (SD: 178 ms) for BDL-COM patients and 832 ms (SD: 178 ms) for controls. Average scores and reaction times as a function of characters (Arrow vs. Avatar) and perspectives (Self vs. Other) were illustrated in Fig. 2. The mixed design ANCOVA on the percentage of correct responses showed an effect of the factor Group (F(1,271) = 8.2, *p* = 0.004) with higher scores for controls, Perspective (F(1,271) = 4.7, *p* = 0.03) with higher scores for Other than Self, a significative interaction between factors Group and Perspective (F(1,271) = 5.6, *p* = 0.02) with lower scores for Self-perspective in BDL-COM patients (Fig. 2A). The covariable Age (F(1,271) = 5, *p* = 0.03) had also a significant effect on the percentage of correct responses with slightly higher scores for older individuals (see also figure in Supplementary Material). There was no significant effect of Character on the percentage of correct responses. The mixed design ANCOVA on the reaction time showed only an effect of the factor Group (F(1,271) = 24.8, *p* = 0.000001) with significantly longer reaction times for BDL-COM patients than controls (Fig. 2B), and of the covariable Age (F(1,271) = 13.6, *p* = 0.0003) with faster response for young individuals (see also figure in Supplementary Material).

**Table 2:** Averages and standard deviations for accuracy and reaction time (RT) in both groups and for the four experimental conditions (arrow with self perspective, arrow with other perspective, avatar with self perspective and avatar with other perspective).

Accuracy		Patients			Controls			
Conditions	Arrow self	Avatar self	Arrow other	Avatar other	Arrow self	Avatar self	Arrow other	Avatar other
Average	89,74	90,57	95,82	95,56	94,33	94,77	95,31	95,37
Std. dev.	13,16	13,20	4,65	5,60	6,46	6,28	5,59	5,65

ANCOVA on accuracy	sum_sq	df	F	PR (> F)	Tuckey HSD			
					mean diff	HSD p-adj	Meaning	
Perspective	224.331752	1.0	4.665766	0.031647	1.7902	0.0341	Self > Other	
Character (Arrow/Avatar)	3.820840	1.0	0.079468	0.778235				
Group	393.818353	1.0	8.190835	0.004539	− 2.0552	0.0459	Controls > Patients	
Perspective × Character	5.894252	1.0	0.122592	0.726512				
Perspective × Group	267.685227	1.0	5.567454	0.019007	− 4.4381; 5.2071; 5.5347	0.0112; 0.0018; 0.0118	Self Controls >self Patients; Self Patients>other controls; Self Patients>other Patients	
Character × Group	0.049833	1.0	0.001036	0.974341				
Perspective × Character × Group	1.275032	1.0	0.026519	0.870761				
Age	239.381546	1.0	4.978779	0.026478				
Residual	13029.779860	271.0	NaN	NaN				

Reaction time	Patients				Controls			
Conditions	Arrow self	Avatar self	Arrow other	Avatar other	Arrow self	Avatar self	Arrow other	Avatar other
Average	1040,07	1033,46	1044,36	1002,14	839,85	828,85	845,37	824,64
Std. dev.	175,42	184,00	177,47	189,51	181,20	177,88	182,91	176,33

ANCOVA on RT	sum_sq	df	F	PR (> F)	Tuckey HSD			
					mean diff	HSD p-adj	Meaning	
Perspective	2.853090e+02	1.0	0.009255	0.923432				
Character	2.228270e+04	1.0	0.722780	0.395984				
Group	7.651621e+05	1.0	24.819433	0.000001	198.0426	0.0	BDL-COM > Controls	
Perspective × Character	4.139749e+03	1.0	0.134280	0.714321				
Perspective × Group	2.525360e+03	1.0	0.081915	0.774938				
Character × Group	8.252643e+02	1.0	0.026769	0.870158				
Perspective × Character × Group	1.954372e+03	1.0	0.063394	0.801401				
Age	4.200795e+05	1.0	13.626049	0.000270				

ANCOVA data include the categorial factors (Perspective, Character, and Group) and Age as confounding variable. The corresponding post hoc analysis for factors and interactions with a significant effect is also reported.

### Scalp level

**Figure 3:**
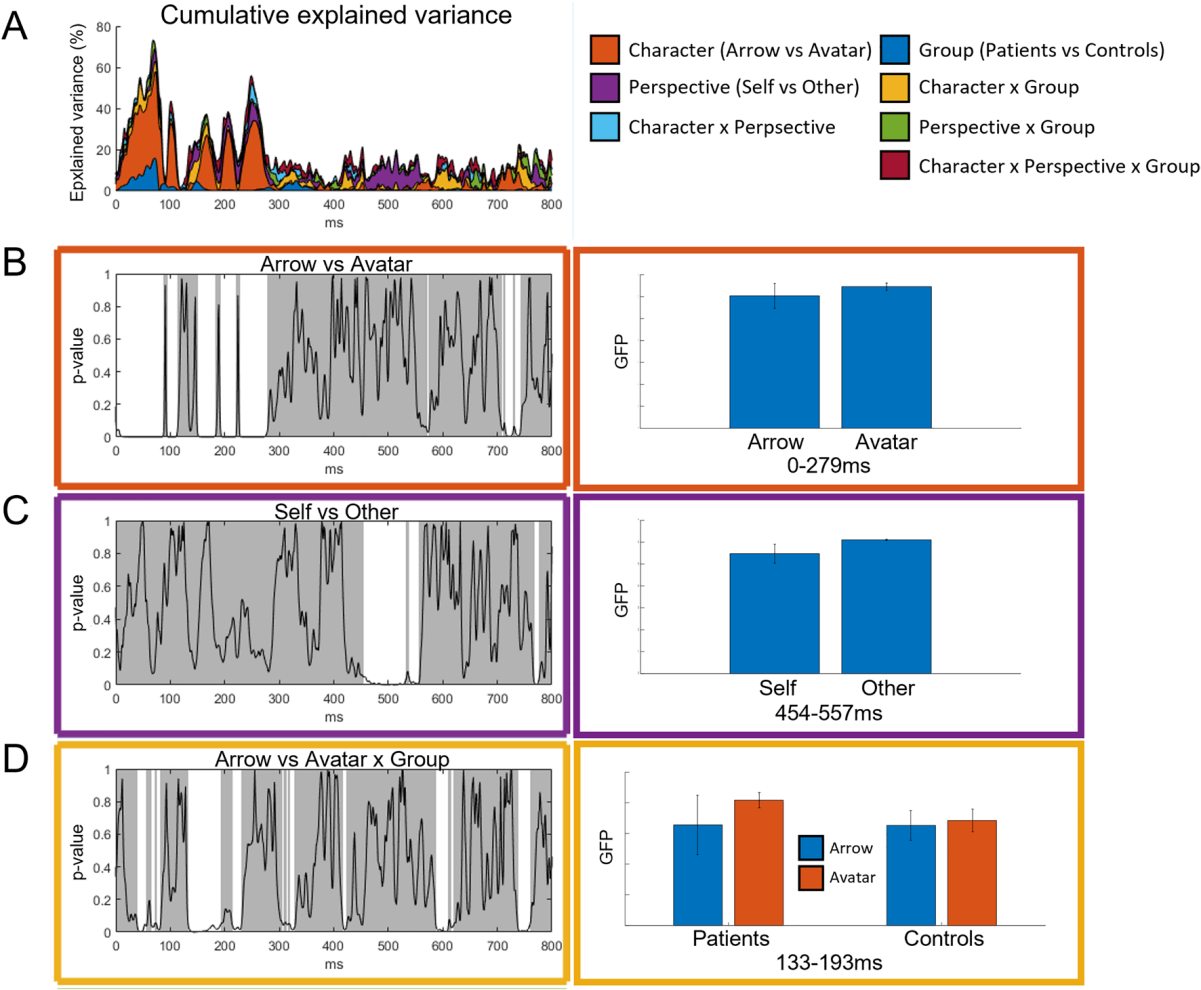
Analysis of EEG global field power. Results of the non-parametric analysis of variance of the GFP according to the Group factor and four different conditions of the task (arrow-avatar × self-other-perspective), and their interaction. (**A**) The upper graph displays the cumulative explained variance for the different factors. (**B**,**C**,**D**) Note that only the factors with significant effects are represented with their respective colour code on the framing. In these graphs, the curve represents the *p*-values and white–grey tones represent the significance across time for each factor, i.e. white for significant time window. The corresponding bar plots and their 95% confidence intervals on the right depict the effects on a noticeable time windows.

Event Related Potentials (ERPs) were computed from 87 epochs in average for patients and 84 epochs in average for controls out of 96 repetitions per condition. Considering the two groups and 4 conditions (Self-Other, Arrow-Avatar), a topographic consistency was observed between 0 and 800 ms post-stimulus, that was more evident for controls compared to the smaller group of patients (Fig. 3). This period was selected for further event related response analyses (scalp level and inverse solution). The ANCOVA analysis on GFP revealed significant period of differences according to the Character (Arrow vs. Avatar) between 0 and 279 ms post-stimulus with a GFP being in average slightly higher for the Avatar condition (*p* = 0.0002; maximum explained variance of 42.21%) (Fig. 3B). GFP values were also significantly higher for the Other compared to Self-perspective between 454 and 557 ms (*p* = 0.009; maximum explained variance of 13.97%) (Fig. 3C). The early GFP difference according to the Group (patients vs. controls) did not reach statistical significance compared to random distribution (*p* = 0.5). There was however a significant interaction between the Group and Character factors (*p* = 0.001; maximum explained variance of 10.56%) with significantly higher GFP for the Avatar and lower GFP for the Arrow between 133 and 193 ms in BDL-COM patients (Fig. 3D).

**Figure 4:**
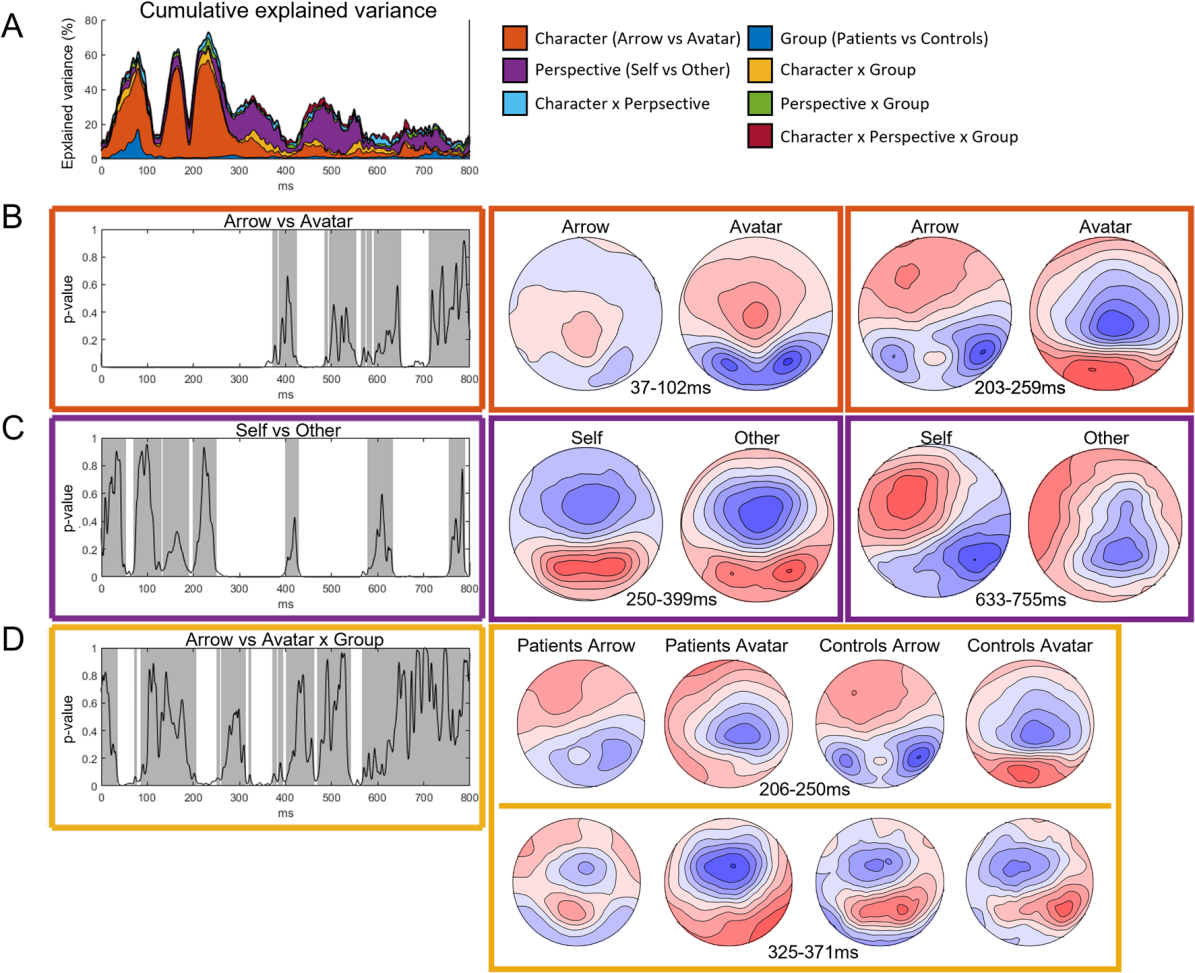
Analysis of EEG topographical differences. Results of the non-parametric analysis of variance of the topographic representation across time according to the Group factor and four different conditions of the task (arrow-avatar × self-other-perspective), and their interaction. (**A**) The upper left graph displays the cumulative explained variance for the different factors. (**B**,**C**,**D**) Note that only the factors with significant effects are represented with their respective colour code on the framing. In these graphs, the curve represents the *p*-values and the white–grey tones represent the significance across time for each factor, i.e. white for significant time window. The topographical maps depict topographic features according to the conditions for the main time windows of interest.

Topographically, the ANCOVA revealed early differences according to the Character factor that were sustained from 0 to 370 ms (*p* = 0.0002; maximum explained variance of 56.29%), and later differences according to the Perspective factor that started at 250 ms post-stimulus (*p* = 0.0002; maximum explained variance of 23.47%) (Fig. 4A–C). The early differences observed for the factor Group and Perspective x Group interaction did not differ from the random distribution. Significant interactions were found between the factors Group and Character occurring intermittently from 37 to 565 ms (*p* = 0.02; maximum explained variance of 6.76%). Controls displayed more pronounced occipito-frontal topographies for the Avatar condition between 50 and 225 ms, then the interactions are driven by patients processing of the Avatar conditions with strong right lateralization between 350 and 550 ms (for illustrations see Fig. 4D).

### Inverse solutions

**Figure 5:**
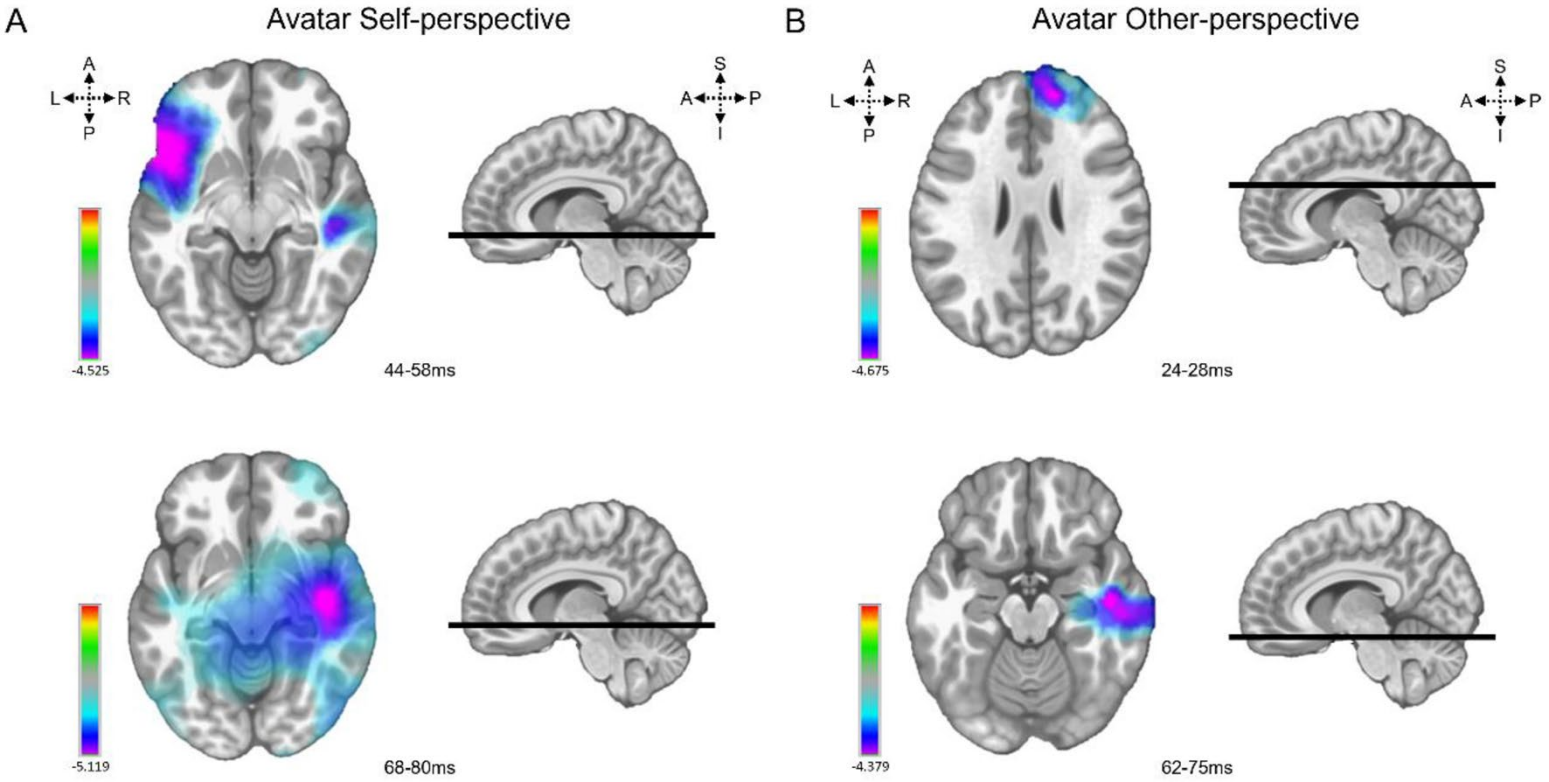
Inverse solutions. Significant differences between BDL-COM and controls from unpaired t-tests on estimated source imaging per condition. (**A**) The right side represents significant group differences in activation patterns for the Avatar and Self-perspective condition. The colours on the brain slices represent the average t-values for significant areas and time windows (cold colours for controls > BDL-COM). (**B**) The left side represents significant group differences in activation patterns for the Avatar and Other-perspective condition.

The comparisons between groups for the Arrow character (Self or Other-perspective) showed only sparse significant differences in the inverse space after FDR correction. However, the two groups differed significantly (*p* < 0.05) and more consistently in activation patterns for the Avatar character. Self-perspective condition was associated with stronger left inferior frontal (BA47) activation from 44 to 58 ms and right middle temporal gyrus and right insula (BA21, 13) activation from 68 to 80 ms in controls compared to patients (Fig. 5A). In the Other-perspective condition, the controls displayed a very short right superior frontal gyrus (BA10) activation from 24 to 28 ms and a right inferior temporal gyrus (BA20), medial superior frontal gyrus with right superior temporal gyrus (BA22) from 38 to 49 ms and fusiform gyrus activation from 64 to 74 ms compared to patients (Fig. 5B).

### Frequency response

**Figure 6:**
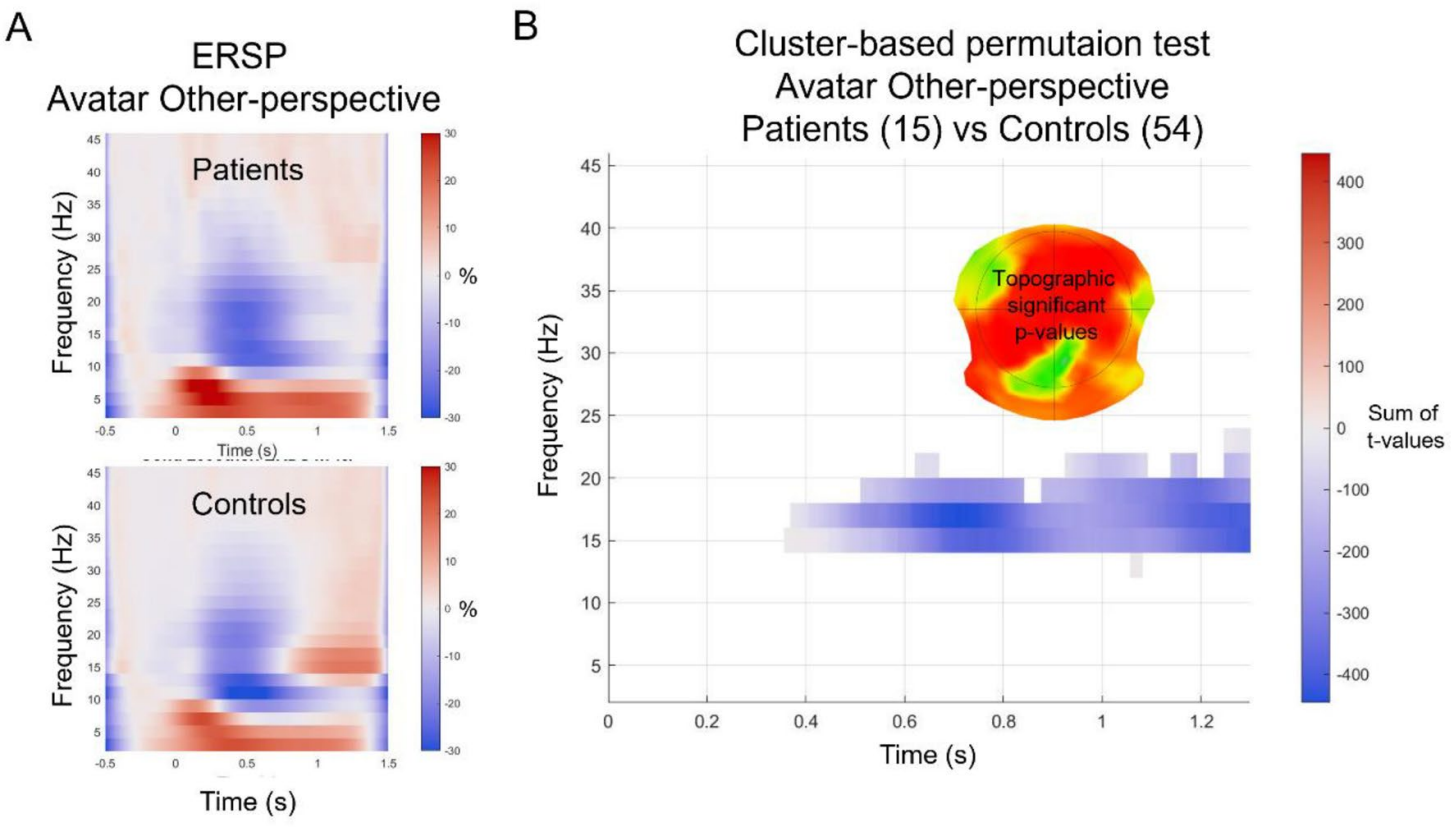
Cluster-based permutation tests on event related frequency decomposition. On the left side (**A**) the graphs show the ERSP for the Avatar and Other-perspective condition for the patients and for the control participants. On the right side (**B**) the graph shows the sum across channels of the t-values (negative blue) along time on x-axis and frequency bins on y-axis for the only significant cluster of the ERSP for the condition Avatar and Other-perspective differing between patients and control participants. The topography represents the corresponding *p*-values as a sum of the significant point across time and frequency bins.

The group comparisons for the ITC did not reveal significant difference for any of the conditions studied. When considering the ERSP, there were significant group differences only for the Avatar character in Other-perspective condition. Controls showed a stronger frequency power in the beta band (14–22 Hz) compared to the patients occurring from 400 to 1300 ms. This cluster of differences was located mainly over the left temporal, parietal and right frontal lobe (Fig. 6).

## Discussion

Using high density EEG, the present data reveals the differential activation of neural generators in dPT between BDL-COM patients and healthy controls. This population of patients is of particular interest when studying the neural substates of empathy deficits in humans since they are known to suffer from increased impulsiveness but also impaired mentalization abilities^33–35^. Our clinical sample includes cases with court-order probation follow-up our court-ordered outpatient treatment after criminal convictions with a main diagnosis of BDL personality disorder. To the best of our knowledge, this is the first study exploring dPT performance in such forensic sample.

Behaviorally, although both controls and BDL-COM patients showed high rates of correct answers in dPT (> 90%), the latter were slightly but significantly less performant mainly when adopting the Self perspective and displayed significantly higher reaction times in all conditions. This observation differs from the results of the only report using the Samson’s dPT in persons with criminal conviction^39^. The authors found that psychopaths display similar performances and reaction times than controls pointing to their ability to manage both the Self and Other perspectives. BDL-COM patients in the present sample displayed higher PCL-R, TAS-20 and HCR-20 scores compared to controls. However, their scores remained modest excluding the presence of clinically overt psychopathy. Our results are in line with several previous reports stressing the vulnerability of BDL patients in terms of emotional expression and moral cognition^32,34,57^. and in particular their increasing difficulties when dealing with the distinction between Self and Other (for review see^58^).

The present findings reveal also significant group differences in the activation of neural generators when treating mentalistic (Avatar) and non-mentalistic (Arrow) stimuli. Several lines of evidence support the idea that BDL-COM patients display significant quantitative and topographic EEG alterations in the Avatar condition compared to controls. First, an increased GFP was observed for this condition in the patient group pointing to the additional brain effort needed for treating this type of stimulus. Second, the activation of brain generators for Avatars, but not Arrows, differs radically between BDL-COM patients and controls, the former showing a late and diffuse right hemisphere involvement contrasting with the well-established occipitofrontal topography reported in controls^25^. As expected, controls display a temporal shifting of brain activation from posterior visual to anterior frontal areas between 50 and 225 ms post-stimulus. In contrast, BDL-COM patients showed a diffuse right hemisphere activation without postero-anterior gradient that occurs in later time points (350 to 550 ms). In inverse solutions the processing of Avatars is also less engaging in BDL-COM patients than controls for both the Self and Other perspective. The adoption of Self perspective for Avatars involves a lower activity of early brain generators in the left inferior frontal, right middle temporal cortex and insula among BDL-COM cases. When judging the Other-perspective, these patients also showed a lower activation of superior frontal, right inferior temporal and fusiform cortex compared to controls within the same time frame. Third, the beta oscillation power was significantly lower in BDL-COM patients compared to controls in late time points post stimulus but only for the Avatar-Other condition. The ERSP bursts of frequency power unlocked to the stimulus in the beta band were more pronounced for the Avatar, but not for Arrow, in controls compared to BDL-COM patients with a strong and widespread beta rhythm response in left temporo-parietal and right frontal areas between 400 and 1300 ms post-stimulus. These observations reveal that BDL-COM patients display both altered topography of dPT-related EEG activation with suppression of the postero-frontal temporal shifting and late and diffuse activation of brain generators in right hemisphere as well as reduced abilities to activate beta oscillations during dPT. They are consistent with previous fMRI reports that documented BPD hypoactivity of cognitive empathy-related areas such as the superior temporal sulcus and the temporoparietal junction when addressing emotional perspective taking^59^ as well as multifaceted empathy tests^60^.

Our data also provide new evidences regarding the patterns of brain activation according to the Self and Other-perspective. The inverse space analysis reveals group differences in the activation of brain generators according to the perspective-taking. Here again, the observed differences concern only Avatars further pointing to the altered brain functionality in BDL-COM patients when facing mentalization processes. Compared to controls, BDL-COM patients showed a decreased activation of brain generators in left inferior frontal, right middle temporal cortex and insula in Self condition that occurs in early time points. This decreased brain activation in Self condition matches with their lower performances in this condition documented by our mixed ANCOVA analysis. In the Other condition, brain activation in frontal and temporoparietal areas was again much less pronounced in BDL-COM patients than controls. This observation is consistent with previous lines of evidence pointing to the difficulty of BDL patients with Self-Other distinction in allocentric perspective^18,61,62^. An increase in beta power may reflect the activation of neural generators needed for top-down processing of sensory information and task prioritization, two cognitive dimensions involved in altercentric judgment^63,64^. Previous reports also pointed to the role of beta oscillations in mentalization processes and revealed significant alterations of this EEG parameter in psychiatric patients^65,66^. The decreased beta power in late time points observed in patients when taking the Other perspective further supports an altered activation of their mentalization-related brain generators.

The present findings are based on high-density EEG and combine topographic analysis, inverse space solutions, and oscillation analysis, both phase and non-phase locked to the stimulus. We focused on forensic patients, a relatively rare clinical sample of cases with documented law violations that implied court-ordered measures. However, there are several limitations to consider. First, in terms of clinical diagnosis, our BDL patients showed low levels of psychopathy. Given the limited number of cases, we were unable to run separate analyses for cases with ADHD, a very frequent comorbidity of this personality disorder. As such, the present sample cannot be thus considered as representative of the full spectrum of BDL-COM patients. The male sample, exclusion of neurological and acute psychiatric disorders, regular use of psychotropics and active substance abuse also limit the generalizability of our observations. Second, the present findings concern the Samson’s dPT which is based on the judgment of visually presented situations and refers to the assessment of observation and inhibition of representation rather than that of beliefs and desires. As such, it is dependent on both attentional processes and implicit mentalization and cannot be considered as representative of the whole ToM spectrum. In the same line, mentalization is not a unique process and its characteristics vary substantially according to the experimental design so the present observations do not allow for drawing general conclusions about the involvement of mentalizing networks in BDL-COM patients. Future studies in larger samples of COM patients including women, a larger diagnostic spectrum (antisocial personality, ADHD) and additional metalizing paradigms, and combining several imaging modalities (fMRI, single photon emission computerized tomography) are warranted to get better insight into the ToM-related deficits in bran activation in forensic samples.

### Supplementary Information

Supplementary Information.

## Data Availability

The raw data supporting these article conclusions will be made available by the correspondence author at vincent.rochas@fcbg.ch, without undue reservation.
