# Supplementary material for: Visual perspective taking neural processing in forensic cases with high density EEG
Source: Sci Rep. 2024 Jul 10;14:15973. doi: 10.1038/s41598-024-66522-y (PMC11237136; doi:10.1038/s41598-024-66522-y)
Supplement: Supplementary file 1 — Supplementary Information. [file 41598_2024_66522_MOESM1_ESM.docx]

**Supplementary material**


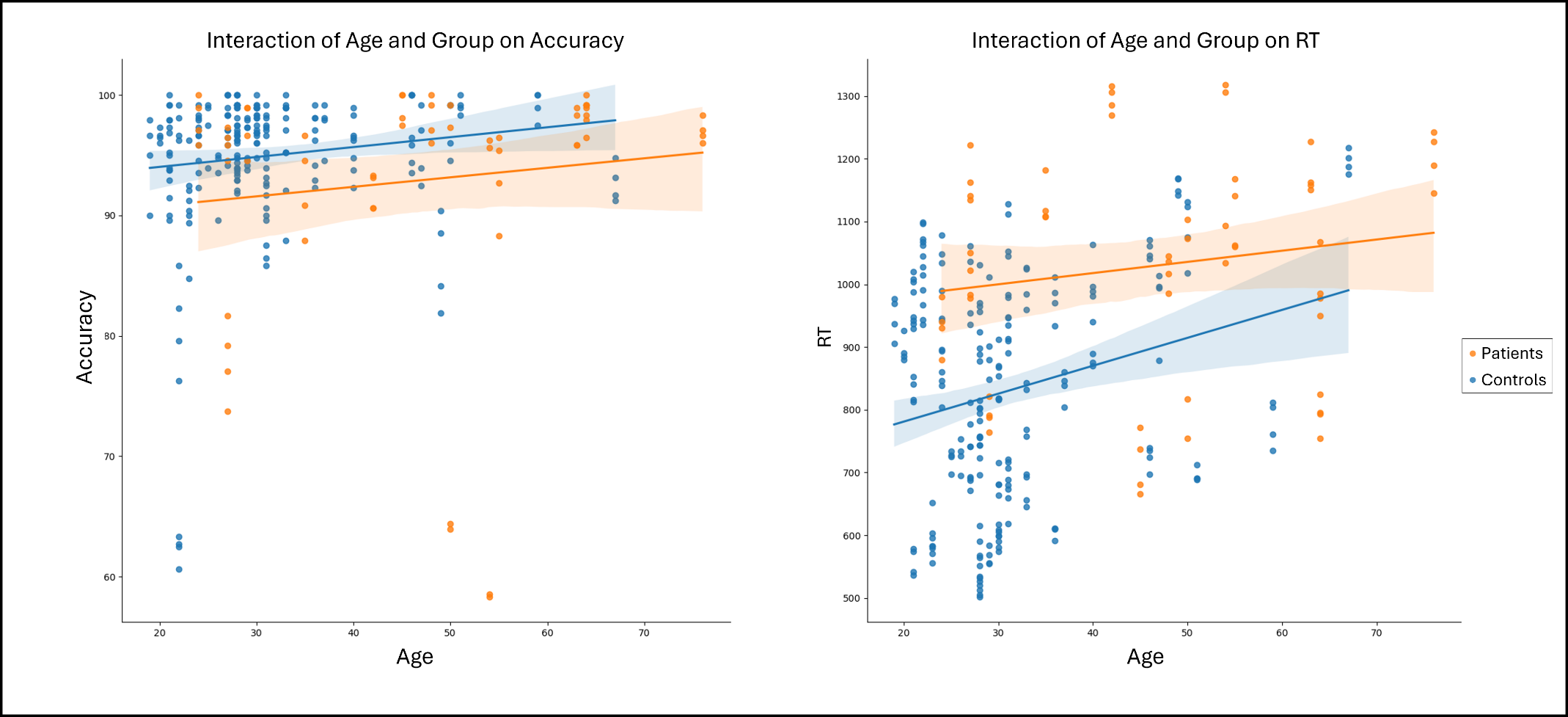


Figure A – Interactions plots of the age effect on the relationship between the factor Group and accuracy, reaction time values (dependent variables). The left plot shows the positive influence of Age on the Accuracy without significant group differences. The right plot shows a clear difference between the groups for the reaction time (RT) with the patient group being less influenced by Age possibly due to a ceiling effect of the RT that is limited by the image presentation time (i.e. 1500ms).
